# Smart marketing may improve public understanding of the anesthesia profession

**DOI:** 10.1186/s13584-015-0011-1

**Published:** 2015-06-15

**Authors:** Barak Cohen, Daniel Ogorek, Stanislav Oifa, Anat Cattan, Idit Matot

**Affiliations:** Division of Anesthesia, Pain and Critical Care, Tel-Aviv Medical Center affiliated with Sackler Medical School, Tel Aviv University, Tel Aviv, Israel

**Keywords:** Public opinion, Anesthesiology, Public campaign

## Abstract

**Background:**

A 2005 survey led by the Israeli Society of Anesthesiologists (ISA) found that large parts of the Israeli public are not familiar with the profession of anesthesia. The ISA has subsequently been conducting a public campaign for several years with the aim to enhance community knowledge regarding the anesthesiologists’ training and their critical role in the perioperative period.

**Objective:**

The present study sought to evaluate the value of a campaign aiming to enhance public understanding of the importance of a medical profession; more specifically, a campaign to promote awareness of the community regarding the anesthesia profession. If proved to be successful, public campaigns may be considered in other countries and for other medical professions with similar difficulties.

**Methods:**

In 2013, five hundred participants from the general community were asked to answer a questionnaire focusing on the profession of anesthesia.

**Results:**

Public knowledge has improved following the campaign. Specifically, improvement was demonstrated regarding the qualification of the anesthesiologist as an MD (92% vs. 64% in 2013 and 2005, respectively), and enhanced awareness of the anesthesia team’s critical role in the operating room (OR) (48% vs. 30% in 2013 and 2005, respectively).

**Conclusions:**

The Israeli community is attentive to public campaigns that address the roles of a medical profession. Enhanced public knowledge regarding the importance of the anesthesia profession may have a significant impact on both the payment policy for anesthesiologists and on the recruitment of more physicians to the field of anesthesia. Public campaigns may be considered for other medical professions with similar difficulties.

## Background

The profession of anesthesiology has long been suffering from low appraisal among the general population. This misperception might have affected the anxiety level of patients who are candidates for surgery [1,2], the medical students or interns who are about to choose their residency [3], and the reimbursement rate for the anesthesiologists [4]. Although many patients are eager to gain information about their upcoming anesthetic management during surgery [1], their actual knowledge regarding anesthesia and its providers is sometimes poor [5-7]. Numerous studies and surveys from around the world have shown that large parts of the public are not aware of the anesthesiologist’s training, duties and responsibilities [8-12]. Moreover, many are not even certain whether the anesthesiologist is a certified MD, and the introduction of the certified registered nurse anesthetist (CRNA) in many countries has added to the confusion.

A survey conducted in 2005 by the Israeli Society of Anesthesiologists (ISA) [4] showed a poor level of knowledge regarding the anesthesia profession among the Israeli public. The survey was conducted as an omnibus telephone survey (collecting answers regarding different issues in the same questionnaire) including 503 adult participants from the general Israeli population; about half were males, the majority was born in Israel, and half had previous surgery. Some of the survey’s main characteristics are shown in Table 1. In that 2005 survey, only 64% of the participants knew that the anesthesiologist is a certified MD, and merely 30% believed that the anesthesiologist is responsible for their well-being during surgery. The survey was conducted in order to better understand the reasons for the unpopularity of anesthesiology as a medical residency among Israeli physicians, and the lack of demand from the public for selection of a private anesthesiologist (as opposed to private surgeon) in the private healthcare market.

**Table 1:** **Data from the 2005 ISA survey**

**Parameter**	**N (% of responses)**
**Survey Characteristics**	Omnibus telephone survey
	503 adult participants
	±4.4% maximal sample error
**Gender**	
Male	247 (49.1)
Female	257 (51.0)
**Past surgery**	
Yes	253 (50.3)
No	249 (49.6)
**Country of birth**	
Israel	328 (65.2)
Other	158 (31.4)
N/A	17 (3.3)
**Does the anesthesiologist have to be an M.D?**	
Yes	323 (64.3)
No	138 (27.4)
Do not know	42 (8.3)
**Who is responsible for the patient’s well-being during surgery?**	
Anesthesiologist	150 (29.9)
Surgeon	212 (42.2)
Nurse	33 (6.6)
Other	82 (16.2)
Do not know	26 (5.1)
**Is the anesthesiologist present in the operating room during the entire operation?**	
Yes	328 (65.2)
No	125 (24.9)
Depends on the type of surgery	12 (2.3)
Other	3 (0.6)
Do not know	35 (7.0)
**In Which of the following sites does the anesthesiologist work?** (you may mark several answers)	
Operating Room	446 (88.7)
Delivery Room	256 (51.0)
Intensive Care Unit	258 (51.4)
Pain Service	147 (29.3)
Emergency Department	179 (35.6)
Invasive procedures	152 (30.2)
Resuscitation	149 (29.7)
Radiology Suite	68 (13.6)
Do not know	23 (4.5)

N/A – not available.

The results of that survey led the ISA to proceed with an intense marketing agenda in the media (newspapers, radio and television) in order to increase public awareness of the various professional aspects of the anesthesia practice. This campaign, mainly directed at the general public, was conducted using different approaches such as paid advertisements, interviews with experts and patients, focused reports and investigations, all of which appeared in the daily newspapers, magazines as well as in the radio and television broadcasts. Additionally, several internet websites were updated and created to fill in for the knowledge gap regarding public awareness of anesthesia risks. Finally, street posters were positioned along major highways and in hospitals in the surgical facility area. The present study was designed to evaluate the current opinions of the Israeli public regarding the anesthesia profession after the conclusion of the ISA campaign.

## Methods

We conducted this descriptive study from March till September 2013. The study was approved by the Institutional Review Board of the Tel Aviv Medical Center, which waived the need for informed consent.

Five hundred questionnaires were handed out in public places (market, gym, shopping mall, university and in hospital) to 500 adult subjects from the general population. Questionnaires were completed anonymously. The questionnaire explored general characteristics of the respondents (age, gender, level of education, country of birth), knowledge of various perspectives of the anesthesia profession and its responsibilities (awareness of the anesthesiologist being an MD, knowledge of the anesthesiologist’s role in the OR, presence during the entire operation, the need for pre-anesthetic evaluation, role in other hospital locations), as well as the level of anxiety from surgery. Lastly, respondents were asked regarding their thoughts as to why there is a shortage of anesthesiologists in Israel.

The survey was pre-tested through patients waiting for pre-anesthetic evaluation and their families. The participants were asked how comprehensive the questions were and to suggest any modification to the questions to improve their comprehension. On the basis of the pretesting, modifications were made to several questions and response categories.

Descriptive statistics were used to analyze the answers to the questionnaires. T- and Chi tests were used where data were compared between subgroups, (male vs. female respondents; those who had previously undergone surgery to those who had not). Results were qualitatively compared to data from the previous ISA survey in 2005, where available.

## Results

Demographic data regarding the 500 participants are shown in Table 2. Nearly 45% of participants were males, and age ranged from 18 to 91 years (mean age 42 years). Two thirds of participants were born in Israel, and about half experienced past surgery. Regarding education, 53% held an academic degree, while 40% graduated high-school or professional school only.

**Table 2:** **Demographic data from the 2013 survey**

**Parameter**	**N (% of responses)**
**Gender**	**479**
Male	214 (44.7)
Female	265 (55.3)
NA	21
**Age, years**	**487**
Mean (SD)	42 (18)
Range	18-91
**Country of birth**	**490**
Israel	331 (67.7)
Other	159 (32.3)
NA	10
**Highest level of education**	**437**
High school	120 (27.5)
Professional school	54 (12.3)
University	232 (53)
Other	31 (7)
NA	63
**Past surgery?**	**497**
Yes	267 (53)
No	230 (46)
N/A	3

N/A – not available.

**Figure 1:**
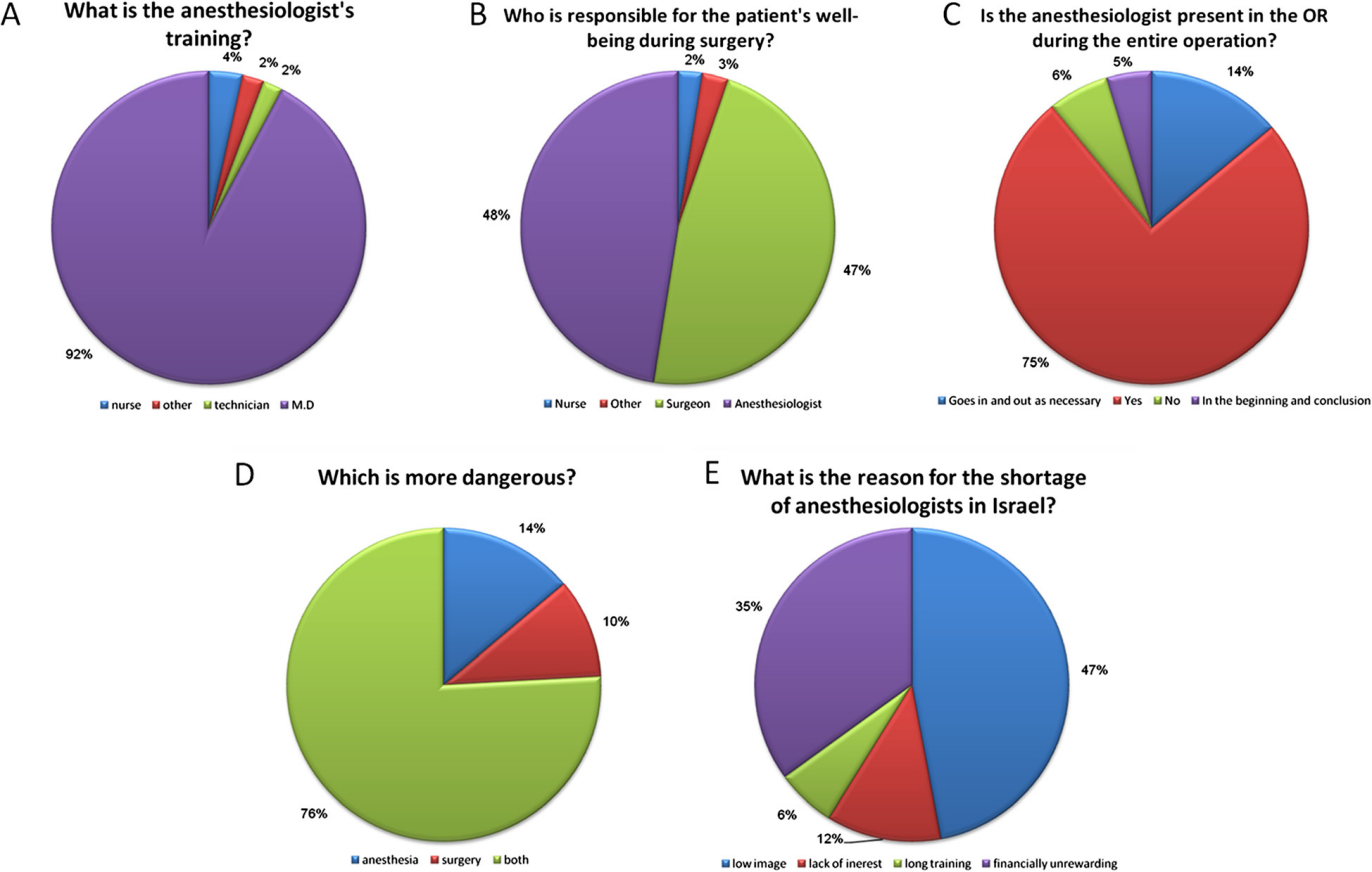
**Answers to selected questions from the survey.** Panels **A**-**E** correspond to the 5 questions mentioned in the figure, see text for details.

The questionnaire and the main results are presented in Table 3 and Figure 1A-E. The vast majority (92%) of respondents knew that the anesthesiologist is an M.D. (Figure 1A), and 48% acknowledged that the anesthesiologist is responsible for their well-being during surgery (Figure 1B). A quarter of the respondents believed that the anesthesiologist does not attend the OR for the whole procedure (Figure 1C).

**Table 3:** **Translated questionnaire with main results**

**Question**	**Number of answers (% of answers)**
**What do you think is the training of an anesthesiologist?**	**492**
Medical Doctor (MD)	454 (92.3)
Nurse	17 (3.5)
Technician	10 (2.0)
Other	11 (2.2)
**Who do you think is responsible for the patient’s well-being during surgery?**	**442**
The surgeon	209 (47.3)
The anesthesiologist	210 (47.5)
The nurse	11 (2.5)
Other	12 (2.7)
**From the list below who do you think it is important to choose ahead of time?**	**500**
The surgeon	141 (28.2)
The anesthesiologist	19 (3.8)
Both	333 (66.6)
Neither	7 (1.4)
**During which stages of surgery do you think the anesthesiologist takes care of the patient?**	**489**
At the beginning of surgery	60 (12.3)
At the conclusion of surgery	0 (0)
Throughout the entire operation	262 (53.6)
In the recovery room after surgery	6 (1.2)
Throughout the entire operation and in the recovery room	161 (32.9)
**Do you think the anesthesiologist is present in the operating room during the entire operation?**	**496**
Yes	373 (75.2)
No	31 (6.3)
The anesthesiologist goes in and out of the operating room as necessary	69 (13.9)
The anesthesiologist provides anesthesia in the beginning of the operation and returns to wake the patient up when surgery is over	23 (4.6)
**What are the anesthesiologist’s responsibilities?**	**489**
To make sure the patient is anesthetized	40 (8.0)
To maintain normal function of the patient’s heart, lungs, brain, kidneys and all other vital organs	15 (3.1)
To wake up the patient at the end of surgery	5 (1.0)
To cooperate with the surgeon	12 (2.5)
All of the above	412 (84.3)
None of the above	5 (1.0)
**In your opinion, which is more dangerous?**	**497**
Surgery	51 (10.3)
Anesthesia	69 (13.9)
Both to the same extent	377 (75.9)
**What, in your opinion, is the reason for the shortage of anesthesiologists in Israel?**	**457**
Lack of interest in this practice	54 (11.8)
Long period of training	28 (6.1)
The practice is financially unrewarding	160 (35.0)
Low image of this profession	215 (47.0)
**In Which of the following sites does the anesthesiologist work? (you may mark several answers)**	**500**
Operating room	490 (98.0)
Recovery room	303 (60.6)
Delivery room	304 (60.8)
Trauma room	193 (38.6)
Pediatric sedation for painful procedures	238 (47.6)
Resuscitations	108 (21.6)
Intensive care unit	230 (46.0)
Pain clinic	169 (33.8)
Pre-anesthesia clinic	309 (61.8)
**On a scale of 1 to 5 (where 1 = no fear at all and 5 = very frightened) how much are you afraid of -**	**mean ± SD**
Surgery (N = 497)	3.47 ± 1.2*
Anesthesia (N = 497)	3.57 ± 1.3*

*Difference between fear of surgery and anesthesia is statistically significant (p < 0.05).

Most participants believed that both anesthesia and surgery are risky to the same extent (76%, Figure 1D), although fear from anesthesia was rated slightly higher on a scale of 1 to 5 than fear from the surgery itself (3.57 ± 1.30 vs. 3.47 ± 1.20, respectively, mean ± SD, t = 2.34, p < 0.05). Greater concern about both anesthesia and surgery was found among women compared to men (t = 8.64, p < 0.01 for surgery and t = 7.22, p < 0.05 for anesthesia) and among participants who had never undergone surgery compared to those who had experienced previous surgery (t = 2.07, p < 0.05 for surgery and t = 2.5, p < 0.05 for anesthesia).

When asked for their thoughts regarding the main reasons leading to a shortage of anesthesiologists in Israel, the most frequent answers were the low image of the profession (47%) and the difficulty in achieving high income (35%) (Figure 1E).

**Figure 2:**
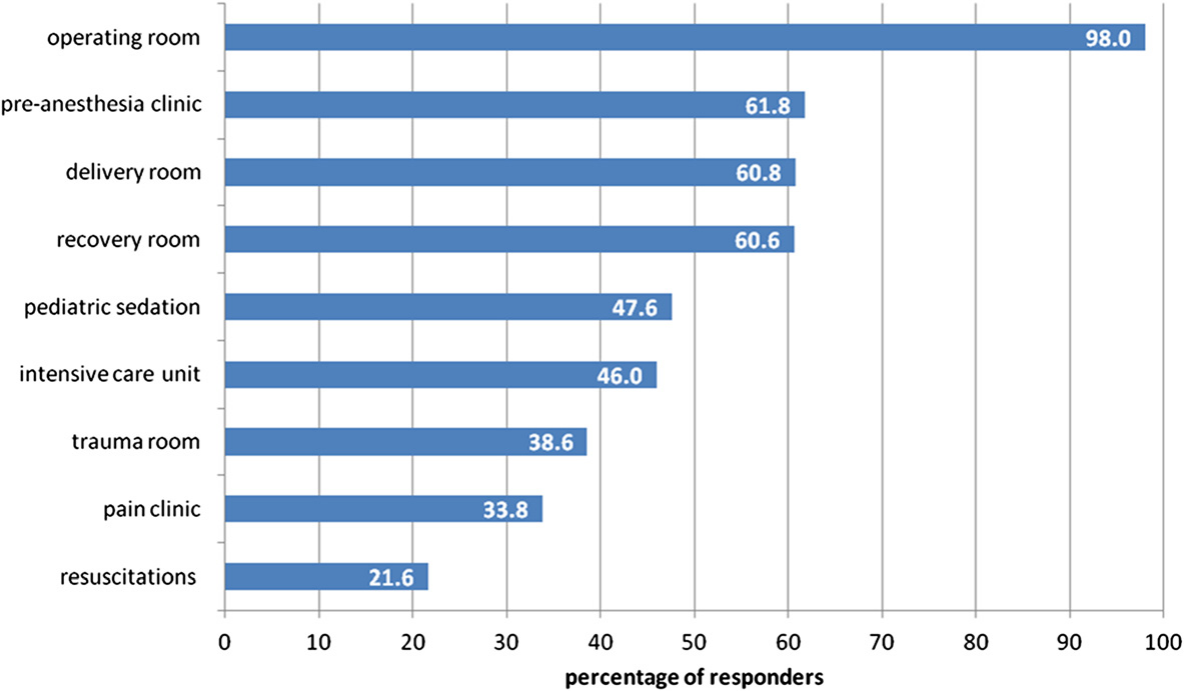
**Public awareness of the anesthesiologist’s working sites.** Percentage of respondents (out of 500) that recognized the different sites in the hospital in which anesthesiologists work.

Almost all (98%) of the respondents were aware of the anesthesiologist’s presence in the OR. Regarding the anesthesiologist’s roles outside the OR, lowest rates of awareness were found concerning the trauma room (39%), pain clinic (34%) and participation in resuscitations (22%), with higher rates for presence in the intensive care units (ICU) (46%), pediatric sedation (48%), recovery room (61%), delivery room (61%) and pre-anesthesia clinics (62%) (Figure 2 and Table 3).

## Discussion

The question regarding the recognition of the anesthesiologist as an M.D is one of the most prevalent in many surveys addressing the issue of public views of anesthesia along the years. Results are diverse, ranging from as low as 58% in some countries [10] to as high as 95% in others [9]. Our survey, performed after 3 years of intensive public media campaign, shows that more than 90% of participants acknowledged that the anesthesiologist is an MD. This result, besides being in line with other publications [8,9,12], represents a significant improvement compared to the previous data from the 2005 survey of the ISA, which reported a rate of only 64%.

Another issue often addressed is the tasks and responsibilities of the anesthesiologist during the operation. Even in surveys in which most participants believe that the anesthesiologist stays in the OR for the whole surgery (75% in our survey), not many know to describe his duties during this period. An Australian survey published in 2007 showed that only 62% knew that the anesthesiologist is in charge of the patient’s vital signs, and as little as 13% knew he is the one dealing with emergencies in the OR [8]. Another survey comparing patients’ knowledge between Australia, Germany and the United states also revealed only partial understanding of the role of the anesthesiologist in dealing with intra-operative problems [10]. In the present study, surgeons and anesthesiologists were rated equally regarding their responsibility for the patient’s intra-operative well-being (47% and 48%, respectively). Again, this represents an improvement compared to the results of the previous Israeli survey in 2005, in which less than a third of the respondents (30%) believed this is the responsibility of the anesthesiologist.

Fear from anesthesia or its possible complications is also a common question, either due to the assumption that people who express fear from anesthesia understand the importance of a competent anesthesiologist, or as means for assessing the efficiency of preoperative anxiety relief by the anesthesiologists. Patients express concern about various aspects of anesthesia, from rare complications such as awareness under anesthesia and failure to awaken at surgery conclusion, to the much more common post-operative nausea, vomiting or pain [2,5]. In the previous ISA survey in 2005, 31% of the respondents thought that anesthesia is riskier than surgery. This estimate almost halved in the present study in which only 14% of respondents were more concerned about the anesthesia than about the surgery. Though hard to draw definite conclusions, it may reflect in part the better understanding of the intraoperative role and tasks of the anesthesiologist, a subject which was thoroughly explained in the campaign.

Roles and duties of the anesthesiologist outside the OR are another common parameter in many surveys, which allows us to compare different populations or different periods in a specific population. Besides the OR, which usually gets the highest rate of acknowledgement (98% in our study), ICU is usually the next prevalent answer to this question. A survey from Finland found that 57% of respondents knew anesthesiologists work in the ICU [12], compared to only 4% from a recent Turkish survey [11], 19% in the Caribbean [13] and up to 71% in Germany [10]. A study from Israel published in 2003 found that only about 5% of respondents knew that anesthesiologists attend the ICU, although the survey conducted by the ISA in 2005 reported a rate higher than 50%, similar to our current result of 46%. Much lower scores are usually reported for participation of anesthesiologists in pain services, as well as resuscitations. Our study is no different in this aspect.

A major part of the ISA campaign addressed the shortage of anesthesiologists in Israel. Results of this shortage were described along the campaign as long waiting periods for surgery, last minute cancelations, and long waiting for labor analgesia. This shortage was vigorously explained by low income and low image of the anesthesiologists. These themes seem to have been disseminated effectively, as more than 80% of participants reported them as the two main reasons for shortage of anesthesiologists in Israel (Figure 1E).

Our study suffers several limitations, including both population and information biases, which are quite common for most publications in this field. Whereas the 2005 survey was conducted by phone using a national sample, the current survey was conducted in person, using a convenience sample in public places (markets, gyms, shopping malls, a university and a hospital) in two large cities. Nevertheless, participants’ populations in the current and previous surveys were similar with respect to gender, country of birth and history of past surgery (Tables 1 and 2).

Another limitation is that, as in previous studies dealing with this topic, there is no control group and results are mostly reported without reference values. Although we did use the ISA 2005 survey as a historical “control” group, the discussed limitations do not allow for quantitative statistical comparison between the results of the two surveys. Results should only be qualitatively compared to assess trends in public awareness to the anesthesia profession. Even a historical comparison between two similar issues might be misleading, since the questions and/or the response options may have been worded, and hence interpreted differently. Different participants might understand the same question in diverse manners – questions addressing the patient’s “well-being” during surgery may be a good example.

It should be mentioned that our study’s results, although comparable in general to those of the original survey of the ISA from 2005, did not use the exact same wording. In addition, participants were not asked about the level of their exposure to the ISA campaign. Hence this study lacks the ability to test the association between the level of subjects’ exposure to the public campaign and the observed effect.

## Conclusion

Public campaigns may be extremely efficient in improving community understanding of anesthesia practice and awareness of the anesthesiologist’s responsibilities. A successful campaign may lead to improvements in the image of a profession and greater desire of young physicians to practice anesthesia [14,15]. In addition, an improved image of the profession among the public could increase the demand for anesthesiologists in private practice and therefore, the ability to improve income. Such promotion might be even more productive in countries where CRNAs have been introduced into the anesthesia practice, a fact that might augment the confusion and decrease knowledge regarding the anesthesiologist’s roles and duties.

Our study shows encouraging results regarding better understanding of anesthesia practice among the Israeli public, compared to a previous survey. These results, although only temporally related to the ISA’s thorough public campaign, suggest that actions directed at increasing public awareness and acknowledgement of medical professions through different media can be very effective. This may prove useful in different countries or other medical professions with similar difficulties.

On-going work is still required in order to better explain the wide scope of this profession to the Israeli public. This would help to alleviate fear among surgical patients, as well as increase the attractiveness of this specialty among medical students and interns. Further study is needed to map the opinions of medical students regarding the anesthesia profession, and the factors that influence these opinions. Results of such studies can direct efforts aimed at increasing the number of anesthesia residents in Israel.
